# Supplementary material for: Epidemiological, Clinical, and Genomic Profile in Head and Neck Cancer Patients and Their Families
Source: Biomedicines. 2022 Dec 17;10(12):3278. doi: 10.3390/biomedicines10123278 (PMC9775590; doi:10.3390/biomedicines10123278)
Supplement: Supplementary file 1 [file biomedicines-10-03278-s001.zip › biomedicines-2078330-supplementary.pdf]

Table S1. Description of tumor sites in 18 index patients (evaluated by array-CGH) and their relatives.

Case	Age/Sex	Tumor site	HPV status	Affected family member /tumor location
2.1	47/M	Buccal mucosa	Negative	sister/breast, uncle/esophagus, other/pancreas
14.1	62/M	Tongue	NA	brother/ oral cavity, sister/lung
26.1	53/M	Soft palate	Negative	mother/uterus, sister/colon
27.1	56/M	Inferior gum	Negative	sister/colon, sister/thyroid
51.1	61/F	Retromolar area	Negative	father/bladder, brother/prostate, brother/HNC, brother/pancreas, brother/esophagus
53	45/M	Pyriform sinus	Negative	mother/breast, aunts/breast
58.1	55/M	Inferior lip	Negative	father/tongue
61.1	68/M	Floor of the mouth	Negative	mother/breast, son/stomach
65.1	63/F	Pyriform sinus	Negative	father/larynx, sister/lymphoma, aunt/colon
66	61/M	Tongue+floor of the mouth+jaw	Negative	brother/kidney sarcoma
67.1	69/M	Tonsil	HPV16	brother/lung
72.1	73/M	Base of tongue	HPV16	brother/esophagus
74.1	51/M	Base of tongue	Negative	father/prostate, brother and uncle/HNC, mother/pancreas, uncle/colon
84.1	49/M	Oropharynx	NA	brother/lymphoma, aunt/breast
168	68/M	Oropharynx	Negative	father/pancreas, cousin/lung
207	74/F	Tongue	Negative	father/melanoma, grandmother/cervical cancer
229	63/M	Floor of the mouth+alveolar edge	Negative	brother/unknown
339	61/M	Floor of the mouth+tongue +gum	Negative	brother/esophagus

HPV: human papilloma virus; HNC: head and neck cancer; NA: not available
